# Cytotoxicity of *Elaoephorbia drupifera* and other Cameroonian medicinal plants against drug sensitive and multidrug resistant cancer cells

**DOI:** 10.1186/1472-6882-13-250

**Published:** 2013-10-02

**Authors:** Victor Kuete, Igor K Voukeng, Roger Tsobou, Armelle T Mbaveng, Benjamin Wiench, Veronique P Beng, Thomas Efferth

**Affiliations:** 1 Department of Pharmaceutical Biology, Institute of Pharmacy and Biochemistry, University of Mainz, Staudinger Weg 5, Mainz 55128, Germany; 2 Department of Biochemistry, Faculty of Science, University of Dschang, Dschang, Cameroon; 3 Department of Plant Biology, Faculty of Science, University of Dschang, Dschang, Cameroon; 4 Department of Organic Biochemistry, Faculty of Science, University of Yaoundé I, Yaoundé, Cameroon

**Keywords:** Cameroon, Cytotoxicity, *Elaoephorbia drupifera*, Medicinal plants

## Abstract

**Background:**

Multidrug resistance (MDR) is a major hurdle for cancer treatment worldwide and accounts for chemotherapy failure in over 90% of patients with metastatic cancer. Evidence of the cytotoxicity of Cameroonian plants against cancer cell lines including MDR phenotypes is been intensively and progressively provided. The present work was therefore designed to evaluate the cytotoxicity of the methanol extracts of twenty-two Cameroonian medicinal plants against sensitive and MDR cancer cell lines.

**Methods:**

The methanol maceration was used to obtain the crude plant extracts whilst the cytotoxicity of the studied extracts was determined using a resazurin reduction assay.

**Results:**

A preliminary assay on leukemia CCRF-CEM cells at 40 μg/mL shows that six of the twenty plant extract were able to enhance less than 50% of the growth proliferation of CCRF-CEM cells. These include *Crinum zeylanicum* (32.22%)*, Entada abyssinica* (34.67%)*, Elaoephorbia drupifera* (35.05%)*, Dioscorea bulbifera* (45.88%)*, Eremomastax speciosa* (46.07%) and *Polistigma thonningii* (45.11%). Among these six plants, *E. drupifera* showed the best activity with IC_50_ values below or around 30 μg/mL against the nine tested cancer cell lines. The lowest IC_50_ value of 8.40 μg/mL was recorded with the extract of *E. drupifera* against MDA-MB231 breast cancer cell line. The IC_50_ values below 10 μg/mL were recorded with the extracts of *E. drupifera* against MDA-MB231 breast cancer cells, *C. zeylanicum* against HCT116 p53^+^/^+^ and HCT116p53^-^/^-^ colon cancer cells and *E. abyssinica* against HCT116 p53^+^/^+^ cells.

**Conclusion:**

The results of the present study provide evidence of the cytotoxic potential of some Cameroonian medicinal plants and a baseline information for the potential use of *Elaoephorbia drupifera* in the treatment of sensitive and drug-resistant cancer cell lines.

## Background

The escape of cancer cells from chemotherapy by multidrug resistance (MDR) mechanisms is until now a major reason for systemic cancer treatment failure. So far, limited progress has been made in the fight against MDR cancer, and even the use of combination chemotherapy cannot solve the problem [1,2]. Medicinal plants and alternative medicine are undeniable sources of new exploitable active principles to manage infectious and degenerative diseases. The structural diversity of chemicals from the medicinal plants makes them valuable tools in the search for potentially active drugs on sensitive and resistant phenotypes. It is estimated that more than 60% of the approved anticancer drugs in the United States of America (from 1983 to 1994) were from natural origin [3,4]. In Cameroon, medicinal plants are traditionally used to manage infectious diseases and different types of cancers [5]. Evidence of the cytotoxicity of these plants against cancer cell lines has been provided [6-11]. In a recent research program, we started to investigate the cytotoxicity of Cameroonian plants against drug-resistant cancer cell lines. The idea is to identify plants able to kill drug-resistant cancer cells with similar efficacy as their drug-sensitive counterparts. Some of the plants identified so far include *Echinops giganteus*, *Imperata cylindrica, Piper capense* and *Xylopia aethiopica* which displayed considerable activities against the P-glycoprotein-expressing adriamycin-resistant cell line, CEM/ADR5000 [7,12]. This encourage us to move forward to search for new cytotoxic agents from Cameroonian medicinal plants, with emphasis on MDR phenotypes with different mechanism of action. The present work was therefore designed to evaluate the cytotoxicity of twenty-two Cameroonian plants against both sensitive and drug-resistant cancer cell lines.

## Methods

### Plant material

All medicinal plants used in the present work were collected at various locations of Dschang, West-Region of Cameroon, between January and April 2012. The plants were identified at the National Herbarium (Yaounde, Cameroon), where voucher specimens were deposited under the reference numbers indicated in Table 1. The air-dried and powdered plant material was soaked in methanol for 48 h, at room temperature. The methanol extract was concentrated under reduced pressure to give the crude extract. This extract was then conserved at 4°C until further use.

**Table 1:** Pharmacognosy of twenty-two studied Cameroonian medicinal plants

**Plant species, family/(Voucher specimen)**^**a**^	**Traditional use**	**Part used traditionally**	**Part used in this study (extraction yield in %)**^**b**^	**Potential active constituents**	**Previously screened activity**
*Ageratum conyzoïdes* Linn. (Asteraceae)/(19050/SFR-Cam)	Purgative, fever, ulcers and wound, mental and infectious diseases, headaches, craw-craw, diarrhea [13].	Leaves, whole plant [13]	Leaves (8.52%)	β-caryophyllene, precocene I, friedelin, Lycopsamine, echinatine,β-sitosterol, stigmasterol, 5-methoxynobiletin, linderoflavone B, eupalestin, sabinene, α and βpinene, 1.6%, β-phellandrene, 1,8-cineole and limonene, ocimene, eugenol [13]	Antimicrobial, anticonvulsant, analgesic, anti-inflammatory, antipyretic, insecticidal [13]
*Albizia gummifera* (Mimosaceae)/(41196/HNC)	Bacterial infections, skin diseases, malaria and stomach pain [14]	Bark	Bark (11.51%)	Vitalboside A, vitalboside-A-2-methylglucuronate, lupeol, lupenone [14]	Antiplasmodial [15]
*Aloe barbadensis* Mill. (Liliaceae), ICNA	Abrasions and burns, emollient and moisturizer [16]	Leaves [16]	Leaves (8.15%)	Aloin A, B, aloesin, aloresin A, aloe-emodin, rhein, aloe-emodin-9-anthrone [16]	Antidiabetic, antiviral, angiogenic, toxicity, immunomodulator [16]
*Cissus quadrangularis* Linn. (Vitaceae)/(18668/SRF-Cam)	Fracture healing, eye diseases, chronic ulcer, tumors, asthma [17]	Stem, pulps [17]	Stem (6.83%)	Alpha and β-amyrin, β-sitosterol, ketosteroid, phenols, tannins, carotene [17]	Antiosteoporotic, analgesic, hypotensive, antibacterial, antifungal [18]
*Crinum zeylanicum* Linn. (Amaryllidaceae)/(18263/SRF-Cam)	Rheumatism, earache, malaria, poison [19]	Bulbs [19]	Whole plant (6.85%)	Flexinine, 6‒hydroxypowelline, zeylamine, hamayne, 3‒acetylhamayne, crinamine, 6‒hydroxycrinamine, 6‒methoxycrinamine, crinine, ambelline, 6‒hydroxybuphandrine, 6‒ethoxybuphandrine, 6‒ethoxybuphanidrine, lycorine, 11‒*O*‒acetoxyambelline, galanthamine, sanguinine, 3‒*O*‒acetylsanguinine [19]	Antiproliferative compounds [19]
*Croton macrostachys* Hochst. (Euphorbiaceae)/(40501/HNC)	Antidiabetic [20]	Roots, bark [20]	Bark (12.72%)	Taraxer-14-en-28-oic acid, trachyloban-19-oic acid, trachyloban-18-oic acid, neoclerodan-5,10-en-19,6β;20,12-diolide, 3α,19-dihydroxytrachylobane, 3α,18,19-trihydoxytrachylobane [20]	Not reported
*Dioscorea bulbifera L* (Dioscoreaceae)/(14274/HNC)	Sore throat and struma, leprosy and tumors, diabetes, microbial infections [21,22]	Rhizome [21]	Rhizome (15.8%)	Kaempferol-3,5-dimethyl ether, caryatin, (+)-catechin, myricetin, quercetin-3-*O*-galactopyranoside, myricetin-3-*O*-galactopyranoside, myricetin-3-*O*-glucopyranoside, diosbulbin B [21]	Analgesic, anti-inflammatory [23], antimicrobial [22]
*Dioscorea dumetorum* (Kunth) Pax Trusted (Dioscoreaceae)/(24431/ SRF-Cam)	Diabetes, topical anesthetic, poison [24]	Leaves	Leaves (6.95%)	Dumetorine, dihydrodioscorine, demethylbatatasin IV, dihydroresveratrol [24,25]	Not reported
*Dissotis perkinsiae* Gilg. (Melastomataceae)/(6991/ SRF-Cam)	Typhoid fever (Personal information)	Leaves, stem	Stem with leaves (10.35%)	Not reported	Not reported
*Elaoephorbia drupifera* (Thonn.) Stapf. (Euphorbiaceae)/(57644/HNC)	Hypertension, diabetes [26]	Leaves [26]	Leaves	Euphol, tirucallol, euphorbol, ingenol elaeophorbate, epitaraxerol, taraxerone, friedelin, lup-20(29)-en-3-one or lupenone, lupeol, olean-12-ene-3-one, olean-12-ene-3-ol,elaeophorbate [27,28]	Leaves extract moderately inhibit HIV-1 and HIV-2 proviral DNA copying [29], relaxant effect on vascular smooth muscles on rats [30]
*Entada abyssinica* Steud. ex A. Rich. (Mimosaceae)/(26967/SRF-Cam)	Bronchitis, coughs, arthritic pain, miscarriage, fever, abdominal pain [31]	Bark, Juice [31]	Bark (13.95%)	Not reported	Not reported
*Eremomastax speciosa* (Hochst) Cufod (Acanthaceae)/(16371/SRF-Cam)	Dysentery, anemia, irregular menstruation, hemorrhoids, urinary tract infection [32]	Stem, leaves [32]	Stem with leaves (8.15%)	Not reported	Anti-diarrhoeal, anti-ulcerogenic [32]
*Gossypium barbadense* L. (Malvaceae)/(25771/HNC)	Cold, bronchitis, palpitations, wounds, systematic diarrhea [33]	leaves, young shoots [33]	Leaves (8.15%)	Gossypol, hemigossypol,6-methoxyhemigossypol, 6-deoxyhemigossypol, 6-methoxygossypol, 6,6′-dimethoxygossypol [34,35]	Antimicrobial [33]
*Kigelia Africana* (Lam.) Benth (Bignoniaceae)/(23220/SRF-Cam)	Dysentery, ringworm, tape-worm, malaria, diabetes, post-partum haemorrhage, pneumonia, toothache, syphilis, gonorrhea [36]	Roots, fruits, leaves, bark [36]	Bark (15.85%)	Pinnatal, norvibutinal, β-sitosterol, 7-hydroxy viteoid II, 7-hydroxy eucommic acid, jiofuran, jioglutolide, kigelin, specioside, verminoside, stigmasterol, lapachol [36]	Antibacterial and antifungal, cytotoxic, analgesic, anti-inflammatory, antimalarial, antiprotozoal, central nervous system stimulant, antidiabetic [36]
(Bignoniaceae)/*Markhamia tomentosa* K.Schum. (1974/SRF-Cam)	Anti-snake venom, sore eyes, heart pain, scrotal elephantiasis [37]	Leaves	Bark (5.31%)	2-acetylnaphtho[2,3-b]furan-4,9-dione, 2-acetyl-6-methoxynaphtho[2,3-b]furan-4,9-dione, oleanolic acid, pomolic acid, 3-acetylpomolic acid, tormentic acid, β-sitosterol, β-sitosterol-3-*O-β*-D-glucopyranoside [38]	Antimicrobial, antiprotozoal [37,38]
Stem and leaves	Malaria [39]	Leaves, roots, stems [39]	Stem with leaves (8.13%)	Paullinoside A, paullinomides A and B, β-amyrin, 13β, 17β-dihydroxy-28-norolean-12-ene, β-sitosterol, β-sitosterol glucopyranoside [40]	Antiparasitic [39]
*Poliostigma thonningii* (Schum.) Milne-Readhead (Caesalpiniaceae)/(32129/HNC)	Leprosy, smallpox, coughs, wounds, ulcers [41]	bark, root, leaves [41]	Bark (13.95%)	Piliostigmin, quercetin, quercitrin, 6-C-methylquercetin 3-methyl ether, 6-C-methylquercetin 3,7,3′-trimethyl ether, 6,8-di-C-methylkaempferol 3-methyl ether, 6,8-di-C-methylkaempferol 3,7-dimethyl ether [42]	Antihelmintic, antitussive, bronchodilator, antibacterial [41]
*Pseudarthria confertiflora* (A. Rich.) Bak. Fabaceae)/(17465/SRF-Cam)	Typhoid fever (Personal information)	Leaves	Leaves (10.75%)	Not reported	Not reported
*Spathodea campanulata* P. Beauv. (Bignoniaceae)/(29470/SRF-Cam)	Kidney diseases, urethra inflammation, poison, enemas, fungus skin diseases, herpes, stomachache, diarrhea [43]	Flowers, leaves, bark [43]	Bark (15.81%)	Ajugol, *p*-hydroxy-benzoic acid, methyl *p*-hydroxy-benzoate [43]	Molluscicidal, hypoglycemic, anti-HIV, anti-malarial [43]
*Spilanthes filicaulis* (Schum et Thonn.) C.D. (Asteraceae)/(42040/HNC)	Toothache, stomach ache, gastritis [44]	Whole plant	Whole plant (5.62%)	Not reported	Analgesic, antimicrobial [45], antiulcerogenic [46]
*Stereospermum acuminatissimum* K. Schum. (Bignoniaceae)/(45705/HNC)	Haemostatic, cicatrizing [47]	Leaves, barks [48]	Bark (13.15%)	1,3,7-trimethylguanin-1/3-ium, 3,7-dimethylguanin-1/3-ium, 2-(4-hydroxyphenyl)ethyl hentriacontanoate, sterequinones A, B, C, E, F, H, zenkequinones A, B, p-coumaric acid, methyl caffeate, caffeic acid, psilalic acid, syringaldehyde, norviburtinal, specioside, verminoside, tyrosol, eutigoside A, ellagic acid, atranorin, ursolic acid, pomolic acid, quinovic acid, oleanolic acid, triacontan-1,30-dioldiferulate, 2-(4-hydroxyphenyl)ethyl dotriacontanoate [47,48]	Antiprotozoal [49]
*Terminalia glaucescens* Planch. (Combretaceae)/(9468/SRFCAM)	Dysentery, AIDS [50]	Bark	Bark (13.84%)	glaucinoic acid, arjunic acid, arjungenin, sericoside, friedelin [50]	Antiplasmodial, cytotoxic, antimicrobial, aldose reductase inhibition [50,51]

^a^Plants were identified at the Cameroon National Herbarium (HNC); ICNA: Voucher with no identification code at the HNC; ^b^The percentage of the methanol extract.

### Extraction

The air-dried and powdered plant samples (1 kg) were soaked in methanol (3 L) for 48 h, at room temperature. The methanol extract was concentrated under *vacuum* to give the crude extract. This extract was then conserved at 4°C until use.

### Chemicals

Doxorubicin, vinblastine and daunorubicin were provided by the University Medical Center of the Johannes Gutenberg University (Mainz, Germany) and dissolved in PBS (Invitrogen, Eggenstein, Germany) at a concentration of 10 mM. Geneticin was purchased from Sigma-Aldrich (Munich, Germany) at a concentration of 50 mg/mL in sterile-filtered H_2_O.

### Cell cultures

Drug-sensitive CCRF-CEM and multidrug-resistant CEM/ADR5000 leukemia cells were maintained in RPMI 1640 medium (Invitrogen) supplemented with 10% fetal calf serum in a humidified 5% CO_2_ atmosphere at 37°C. Sensitive and resistant cells were kindly provided by Dr. Axel Sauerbrey (Department of Pediatrics, University of Jena, Jena, Germany). The generation of the resistant subline was previously described [52]. The specific overexpression of P-glycorprotein, but not other ABC transporters has been reported [53,54]. Breast cancer cells, transduced with control vector (MDA-MB-231-pcDNA3) or with cDNA for the breast cancer resistance protein *BCRP* (MDA-MB-231-*BCRP* clone 23), were maintained under standard conditions as described above for CCRF-CEM cells. Human wild-type HCT116 (*p53*^*+/+*^) colon cancer cells as well as knockout clones HCT116 (*p53*^*-/-*^) derived by homologous recombination were a generous gift from Dr. B. Vogelstein and H. Hermeking (Howard Hughes Medical Institute, Baltimore, MD). Human glioblastoma multiforme U87MG cells (non-transduced) and U87MG cell line transduced with an expression vector harboring an epidermal growth factor receptor (*EGFR*) gene with a genomic deletion of exons 2 through 7 (U87MG.Δ*EGFR*) were kindly provided by Dr. W. K. Cavenee (Ludwig Institute for Cancer Research, San Diego, CA) [55]. MDA-MB-231-*BCRP,* U87MG.Δ*EGFR* and HCT116 *(p53*^*-/-*^*)* were maintained in DMEM medium containing 10% FBS (Invitrogen) and 1% penicillin (100 U/mL)-streptomycin (100 μg/mL) (Invitrogen) and were continuously treated with 800 ng/mL and 400 μg/mL geneticin, respectively. Human HepG2 hepatocellular carcinoma cells and normal AML12 heptocytes were obtained from the American Type Culture Collection (ATCC, USA). The above medium without geneticin was used to maintain MDA-MB-231, U87MG, HCT116 (*p53*^*+/+*^), HepG2 and AML12 cell lines. The cells were passaged twice weekly. All experiments were performed with cells in the logarithmic growth phase.

### Resazurin reduction assay

Resazurin reduction assay [56] was performed to assess the cytotoxicity of the studied samples toward various sensitive and resistant cancer cell lines. The assay is based on the reduction of resazurin, to the highly fluorescent resorufin by viable cells. Non-viable cells rapidly lose the metabolic capacity to reduce resazurin and thus produce no fluorescent signal. Briefly, adherent cells were detached by treatment with 0.25% trypsin/EDTA (Invitrogen, Darmstadt, Germany) and an aliquot of 1 × 10^4^ cells was placed in each well of a 96-well cell culture plate (Thermo Scientific, Langenselbold, Germany) in a total volume of 200 μL. Cells were allowed to attach overnight and then treated with different concentrations of the studied sample. For suspension cells, aliquots of 2 × 10^4^ cells per well were seeded in 96-well-plates in a total volume of 100 μL. The studied sample was immediately added in varying concentrations in an additional 100 μL of culture medium to obtain a total volume of 200 μL/well. After 24 h or 48 h, 20 μL resazurin (Sigma-Aldrich, Schnelldorf, Germany) 0.01% w/v in double-distilled water (ddH_2_O) were added to each well and the plates incubated at 37°C for 4 h. Fluorescence was measured on an Infinite M2000 Pro™ plate reader (Tecan, Crailsheim, Germany) using an excitation wavelength of 544 nm and an emission wavelength of 590 nm. Each assay was done at least two times, with six replicate each. The viability was evaluated based on a comparison with untreated cells. IC_50_ values represent the sample’s concentrations required to inhibit 50% of cell proliferation and were calculated from a calibration curve by linear regression using Microsoft Excel.

## Results and discussion

**Figure 1:**
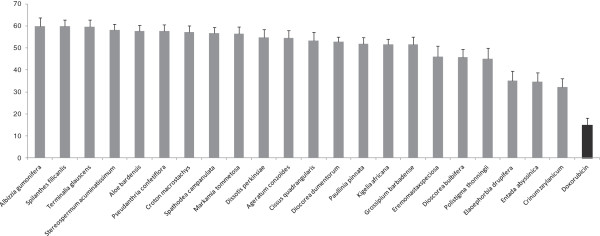
Growth percentage (%) of leukemia CCRF-CEM cancer cell line treated with plant extracts at 40 μg/mL and doxorubicin (10 μg/mL).

In a prescreening of twenty-two plants, we tested a single concentration of 40 μg/mL for each sample against the sensitive CCRF-CEM leukemia cell line. The results depicted in Figure 1 indicate that six of the twenty-two plant extracts were able to display less than 50% growth proliferation of CCRF-CEM cells. These include *Crinum zeylanicum* (32.22%)*, Entada abyssinica* (34.67%)*, Elaoephorbia drupifera* (35.05%)*, Dioscorea bulbifera* (45.88%)*, Eremomastax speciosa* (46.07%) and *Polistigma thonningii* (45.11%). The IC_50_ values of these samples were then determined on a panel of cancer cell lines, including both sensitive and MDR phenotypes. The results are shown in Table 2. Only the *Elaoephorbia drupifera* extract as well as the control drug doxorubucin inhibited the proliferation of the nine studied cancer cell lines, with IC_50_ values below 40 μg/mL. Other extracts showed selective activities, the IC_50_ values being obtained on 6/10 tested cells lines for *Crinum zeylanicum*, 4/10 for *Dioscorea bulbifera* and *Entada abyssinica*, 3/10 for *Eremomastax speciosa* and *Polistigma thonningii* (Table 2)*.* According to the criteria of the American National Cancer Institute, 30 μg/mL is the upper IC_50_ limit considered promising for purification of a crude extract [57]. Consequently, the highest concentration tested (40 μg/mL) in our screening was slightly above this limit. Considering this cutoff point, the IC_50_ values below or around 30 μg/mL were recorded with only the *E. drupifera* extract against the nine tested cancer cell lines (Table 2). However, other extract also displayed activities with IC_50_ values below 30 μg/mL on at least one of the cancer cell line tested.

**Table 2:** Cytotoxicity of the studied extracts towards sensitive and drug-resistant cancer cell lines and normal cells as determined by the resazurin assay

**Cell lines**	**Samples, IC**_**50**_**values (μg/mL) and degree of resistance**^**a**^**(in brakets)**						
	***Crinum zeylanicum***	***Dioscorea bulbifera***	***Elaoephorbia drupifera***	***Entada abyssinica***	***Eremomastax speciosa***	***Polistigma thonningii***	**Doxorubucin**
CCRF-CEM	17.22 ± 2.19	19.77 ± 2.22	11.86 ± 1.19	15.81 ± 1.47	23.65 ± 2.51	26.44 ± 1.18	0.11 ± 0.01
CEM/ADR5000	23.67 ± 1.97 (1.38)	- (>2.02)	13.72 ± 0.97(1.12)	- (>2.53)	38.71 ± 4.12 (1.64)	- (>1.51)	195.12 ± 14.30 (1772)
MDA-MB231	18.01 ± 1.61	33.17 ± 2.91	8.40 ± 0.55	29.14 ± 1.83	35.13 ± 2.49	34.19 ± 2.24	1.10 ± 0.01
MDA-MB231*/BCRP*	11.18 ± 1.11 (0.62)	- (>1.21)	30.96 ± 2.74(3.69)	- (>1.37)	- (>1.14)	- (>1.17)	7.83 ± 0.01 (7.11)
HCT116 *(p53*^*+/+*^*)*	4.32 ± 0.52	36.14 ± 2.37	25.36 ± 2.13	9.55 ± 1.11	-	-	1.43 ± 0.02
HCT116 *(p53*^*-/-*^*)*	7.45 ± 0.64 (1.73)	- (<0.69)	28.61 ± 3.08(1.13)	14.38 ± 1.25 (1.51)	-	-	4.06 ± 0.04 (2.84)
U87MG	-	-	23.58 ± 2.20	-	-	34.22 ± 2.74	1.06 ± 0.03
U87MG*.ΔEGFR*	-	27.76 ± 1.86(<0.69)	16.03 ± 0.88(0.68)	-	-	- (>1.17)	6.11 ± 0.04 (5.76)
Hep-G2	-	-	23.23 ± 1.67 (1.72)	-	-	-	1.41 ± 0.12 (<0.04)
AML12	-	-	-	-	-	-	-

^a^The degree of resistance (in brakets) was determined as the ratio of IC_50_ value of the resistant/IC_50_ sensitive cell line; (-): >40 μg/mL.

MDR is a major hurdle for cancer treatment worldwide and accounts for chemotherapy failure in over 90% of patients with metastatic cancer [1,58]. In the present work, we investigated both sensitive and MDR cell lines. The degrees of resistance were calculated by dividing the IC_50_ value of the resistant cell line by the corresponding parental sensitive cell line. We tested cell models overexpressing two ATP-binding cassette transporters, *i.e.* P-glycoprotein (ABCB1/MDR1) or breast cancer resistance protein (ABCG2/BCRP). Furthermore, we tested a p53 knockout cell line and a transfectant cell line harboring a mutation-activated *EGFR* gene (*ΔEGFR*) as examples for resistance-inducing tumor suppressors and oncogenes. Finally, we investigated HepG2 liver cancer cells and AML12 normal hepatocytes to compare carcinoma cells with normal cells. The degree of resistance on the tested cell line toward the control drug doxorubicin was generally high, showing that the studied cell lines can obviously be considered as suitable cell models to study drug resistance. For the most active extract *E. drupifera*, it can be observed that the degrees of resistance were in all cases lower than those of doxorubicin, suggesting that this sample can be exploited in a possible fight against cancer diseases involving MDR phenotypes. In addition, collateral sensitivity (sample more active on resistant cells than on sensitive cells) was observed with the extract of *E. drupifera* against U87MG.*ΔEGFR*, highlighting its good antiproliferative activity.

To the best of our knowledge, the cytotoxicity of the six most active extracts (*C. zeylanicum, D. bulbifera, E. drupifera, E. abyssinica, E. speciosa* and *P. thonningii*) is being reported for the first time. Nevertheless, compounds with activities against malignant cells such as crinine, 6-hydroxybuphanidrine and 6-ethoxybuphanidrine were isolated from *C. zeylanicum*[19]. Also, lupeol [27,28] a moderately active cytotoxic compound [59] was identified in *E. drupifera*, the plant that displayed the best activity as observed in this study. The presence of such compounds could probably explain their antiproliferative activity.

## Conclusion

In conclusion, the results of the present study provide evidence of the cytotoxic potential of some Cameroonian medicinal plants and highlight the good activity of *Elaoephorbia drupifera* on sensitive and drug-resistant cancer cell lines. This plant is a potential cytotoxic source, that could be explored in more details in the future to develop novel anticancer drugs against sensitive and resistant phenotypes.

## Competing interest

The authors declare that they have no competing interests.

## Authors’ contributions

VK*,* IKV, ATM, RT, BW, and VPB carried out the study; VK and TE designed the experiments. VK wrote the manuscript; VK and TE supervised the work. All authors read and approved the final manuscript.

## Pre-publication history

The pre-publication history for this paper can be accessed here:

http://www.biomedcentral.com/1472-6882/13/250/prepub
